# The Association of Corticosteroid Therapy With Mortality and Length of Stay Among Children With Septic Shock: A Retrospective Cohort Study

**DOI:** 10.7759/cureus.33267

**Published:** 2023-01-02

**Authors:** Hamad A Alkhalaf, Nawaf A Alhamied, Abdulmajeed M Alqahtani, Faisal A Alsomali, Malek A Alrasheed, Mohammed M Alhafi, Muhannad Q Alqirnas, Fawaz A Alhamied, Faris M Albaqami, Abdulaziz S Almosa, Fatmah Othman, Mohammed Naeem

**Affiliations:** 1 Department of Pediatrics, King Abdullah Specialized Children Hospital, King Abdulaziz Medical City, Ministry of National Guard Health Affairs, Riyadh, SAU; 2 King Abdullah International Medical Research Center, King Abdulaziz Medical City, Ministry of National Guard Health Affairs, Riyadh, SAU; 3 College of Medicine, King Saud Bin Abdulaziz University for Health Sciences, Riyadh, SAU; 4 College of Medicine, King Saud bin Abdulaziz University for Health Sciences, Riyadh, SAU; 5 College of Public Health and Health Informatics, King Saud Bin Abdulaziz University for Health Sciences, Riyadh, SAU; 6 Intensive Care Unit, King Abdullah Specialized Children Hospital, King Abdulaziz Medical City, Ministry of National Guard Health Affairs, Riyadh, SAU

**Keywords:** septic shock, corticosteroids, sepsis, saudi arabia, peditric sepsis, severe sepsis

## Abstract

Introduction

Septic shock remains a leading cause of mortality in pediatric patients. Corticosteroids have been used in the management of sepsis and septic shock, but there is conflicting evidence on the potential benefit of corticosteroid therapy. This study assessed the risk of mortality and length of stay in the pediatric intensive care unit (PICU) among pediatric patients admitted with a septic shock diagnosis.

Method

A retrospective cohort study was conducted among pediatric patients (up to 14 years old) admitted with a septic shock diagnosis to the PICU of King Abdullah Specialist Children's Hospital in Riyadh from January 2016 to December 2021. The clinical outcomes of patients receiving corticosteroid therapy were compared to those of control patients who were not given corticosteroids. Electronic medical records provided clinical data, severity scores, and the management given for each patient. The patients were followed up from the date of sepsis diagnosis to hospital discharge. Proportional hazard ratios (HRs) were calculated to compare the risk of mortality, length of PICU stay, and length of hospital stay.

Results

A total of 182 pediatric patients were included in the study, and 86 (47%) received corticosteroid therapy. The median age of the study population was 15 months (interquartile range [IQR]: 2-72 months). Compared to the controls, the patients who received corticosteroids had a higher total Sequential Organ Failure Assessment (SOFA) score (mean±SD: 5.5±3 vs. 7.1±3.3, respectively; p <0.01) and required more ventilation support (72% vs. 28%, respectively) and the use of inotropes and vasopressors (74% vs. 34% and 32% vs. 6%, respectively). In-hospital mortality did not significantly differ between the groups (adjusted HR: 2.66; 95% confidence interval [CI]: 0.66-10.28). Those patients who received corticosteroids had 42% less risk of staying in the PICU for over six days than those not receiving steroids (HR: 0.35; 95% CI: 0.13-0.98)

Conclusion

After adjusting for baseline characteristics, severity scores, and medical intervention, no association was found between receiving corticosteroids and mortality (p=0.492). Furthermore, patients who received corticosteroids had less risk of a prolonged stay in the PICU than those who did not.

## Introduction

Sepsis is an extreme, dysregulated bodily reaction to an infection that occurs when a pre-existing infection initiates a response that involves the whole body, a systemic response [1]. It is mostly associated with infections that involve the lungs, gastrointestinal tract, and urinary tract. When sepsis is not treated promptly, it can quickly lead to major, life-threatening impacts, including tissue damage, organ failure, and death. Patients with sepsis require hospital admission, close monitoring, and treatment as quickly as possible [2]. The literature on the management of pediatric sepsis is largely extrapolated from adult sepsis studies, and only recently have preliminary prospective pediatric and sepsis studies been undertaken [3]. The treatment of sepsis comprises resuscitative therapy and antibiotics, but the role of steroids is controversial [4].

A recent study in a local cohort found that the mortality rate for severe sepsis was 16.9% [5]. Regarding the frequency of steroid use in sepsis, a Canadian multi-center study concluded that steroids were administered in 22.3% of all patients with septic shock and in 59.4% of those with refractory shock. Hydrocortisone use was also associated with increased time on vasopressors and incidence of positive cultures [6]. Another meta-analysis reviewed 42 randomized control trials (RCTs) involving 10,194 patients and found that steroid use in cases of sepsis may slightly reduce or not reduce the relative risk of death in the short term (28-31 days) and possibly yield a small improvement in long-term mortality (60 days - one year). Corticosteroids probably also slightly reduce the length of stay in the ICU. Furthermore, hydrocortisone is likely to increase the risk of hypernatremia, hyperglycemia, and neuromuscular weakness. In critically ill patients with sepsis, corticosteroids possibly result in a small reduction in mortality while also possibly increasing the risk of neuromuscular weakness [7]. The literature on this topic also describes a higher mortality rate for pediatric septic patients on steroids, but this increase in mortality can be attributed to the fact that steroids are administered in more severely ill children [8].

In Saudi Arabia, there are very few published studies on pediatric sepsis, and, to our knowledge, none has reported the predictive factors for a favorable response to corticosteroid therapy in our population. We assessed the favorable factors for steroid use in pediatric sepsis and investigated the association between hydrocortisone administration timing and morbidity and mortality in pediatric sepsis. We believe that our findings will promote a better understanding of steroid use in pediatric sepsis and subsequently improve disease outcomes.

## Materials and methods

Study design and setting

A retrospective cohort study was conducted at King Abdullah Specialist Children's Hospital (KASCH) in Riyadh from January 2016 through December 2021. KASCH is an academic tertiary care hospital with an inpatient bed capacity of 220 and 25 beds assigned to the medical/surgical pediatric intensive care unit (PICU). The hospital provides all types of care to employees of the Ministry of National Guard Health Affairs (NGHA) and their families, from primary health care through tertiary specialized care.

Study population and outcomes

All eligible pediatric patients aged 0-14 years who were admitted to the PICU during the study period with a diagnosis of sepsis or septic shock were identified consecutively using electronic medical file data. Patients were identified with sepsis if they presented a life-threatening organ dysfunction due to dysregulated host response to infection; organ dysfunction was defined as an acute change in the total Sequential Organ Failure Assessment (SOFA) score of two points or greater secondary to the infection cause [9].

The following definition was followed in identifying septic shock: persistent hypotension requiring vasopressors to maintain a mean arterial pressure of 65 mm Hg or higher and a serum lactate level greater than 2 mmol/L (18 mg/dL) despite an adequate volume of resuscitation [9]. Patients older than 14 years, who received long-term corticosteroid or other immunosuppressive drugs prior to admission date, or who had incomplete data were excluded from the study. The data collected for the sepsis definition was validated and reviewed by two pediatric consultants.

Standard therapy was provided to all patients admitted to the PICU in terms of antibiotics, inotropic or vasopressor therapy, fluid replacement therapy, corticosteroid therapy, or mechanical ventilation. Based on the corticosteroid management of each patient, patients who received any intravenous corticosteroid were placed in the corticosteroids group (exposed), and those who did not were assigned to the non-corticosteroid group (unexposed).

Data were collected from the medical files regarding patient demographics, pre-existing comorbidities, the presence of central lines, the route of infection, admission laboratory results, and causative organisms. Severity score information was collected using the SOFA, the Pediatric Logistic Organ Dysfunction (PELOD), and the Glasco Coma Scale (GCS) instruments. The primary outcome of the study was hospital mortality from the date of sepsis diagnosis (index date). The secondary outcome was the length of stay in the PICU (defined from the date of sepsis diagnosis to PICU discharge or death, whichever was earliest).

Ethical approval was granted by the institutional review board committee of King Abdullah International Medical Research Center at the Ministry of National Guard Health Affairs on 5 February 2022 (protocol number: NRC22R/012/01). This approval confirmed adherence to the principles of the Declaration of Helsinki.

Statistical analysis

Descriptive analysis was conducted for continuous variables, which are expressed as mean and standard deviation (SD) for normally distributed variables and as median and interquartile range (IQR) if otherwise. For the categorical variables, we used frequency and percentage. Based on their corticosteroid exposure, the patients were assigned to one of two exposure groups. Chi-square and t-tests were used to measure the association between patient variables and exposure groups, and the Mann-Whitney U test was conducted for skewed data. Cox proportional-hazards modeling was employed to estimate the hazard ratio (HR) for outcomes in those receiving corticosteroids; the results were adjusted for potential confounders with a 95% confidence interval (CI). The index time was defined as the time of sepsis diagnosis for both the exposed and unexposed groups. The two cohorts were followed from the index date until the earliest of the following: death date, discharge date from the hospital, or date of study termination. The validity of the proportional-hazards assumption was tested by plotting log-minus-log survival curves and carrying out Schoenfeld tests. A potential confounder was included in the model if it modified the HR by more than 10%. Missing data were categorized into a separate category. Secondary analyses were performed for mortality within 28 days of the index date, for the length of PICU stay, and for the length of hospital stay; the participants were assigned to two categories based on their median PICU stay and median hospital stay. A p-value of less than 0.05 was established as statistically significant. All analyses were done with Stata 12 (StataCorp LLC, College Station, Texas, USA).

## Results

Description of the study population by exposure status

During the study period, a total of 182 patients met the eligibility criteria and were enrolled in the study. Of the septic patients, 43.9% were less than one year old, 57.6% were male, and 76.3% had pre-existing medical conditions at admission, the most common was neurological diseases (26.3%), followed by genetic/metabolic diseases (21.4%). The most common primary site of infection was the respiratory tract (48.3%), followed by bloodstream infection (12.6%). The average SOFA score at admission was 6.3 (SD=3.2), whereas the average PELOD score was 5.5 (SD=4; Table 1).

**Table 1:** Descriptive characteristics of the study sample of patients admitted to the pediatric intensive care GCS - Glasco Coma Scale; IQR - interquartile range; SD - standard deviation; SOFA - Sequential Organ Failure Assessment; PELOD - Pediatric Logistic Organ Dysfunction; VIS - vasoactive-inotropic score The percentage in the table is presented as a column percentage. * Significant p-value

Variables	All patients (n=182)	Non-corticosteroids group (n=96, 52.7%)	Corticosteroids group (n=86, 47.3%)	p-value
Age in months (median, IQR)	15 (2-72)	12 (2-84)	17 (2-72)	0.801
Age groups				0.631
<1 year	80 (43.9)	44 (45.8)	36 (41.9)	
1 year < 6 years	51 (28.0)	24 (25.0)	27 (31.4)	
>6 years	51 (28.1)	28 (29.2)	23 (26.7)	
Gender				0.432
Male	105 (57.7)	58 (60.4)	47 (54.7)	
Female	77 (42.3)	38 (39.6)	39 (45.3)	
Pre-existing medical condition at admission				0.007*
Non	43 (23.6)	15 (15.6)	28 (32.6)	
Yes	139 (76.4)	81 (84.4)	58 (67.4)	
Comorbidity system among pre-existing medical condition at admission				
Neurology disease	48 (26.3)	30 (31.2)	18 (20.9)	0.115
Genetic/metabolic disease	39 (21.4)	24 (25.0)	15 (17.4)	0.215
Cardiovascular disease	5 (2.7)	3 (3.1)	2 (2.3)	0.742
Pulmonary disease	14 (7.9)	2 (2.1)	12 (13.9)	0.003*
Malignancy	10 (5.4)	8 (8.3)	2 (2.3)	0.076
Genitourinary	8 (4.4)	6 (6.2)	2 (2.3)	0.197
Gastrointestinal	10 (5.4)	6 (6.2)	4 (4.6)	0.637
Other	5(2.7)	2 (2.1)	3 (3.9)	0.563
Have central line	76 (41.7)	20 (20.8)	56 (65.1)	<0.001*
Jugular	27 (35.1)	7 (33.3)	20 (35.7)	
Subclavian	15 (19.4)	6 (28.5)	9 (16.1)	
Femoral	34 (44.7)	7 (35.0)	27 (48.2)	
The primary site of infection				0.099
Bloodstream infection	23 (12.6)	16 (16.6)	7 (8.1)	
Respiratory tract	88 (48.3)	37 (38.5)	51 (59.3)	
Central nervous system	19 (10.4)	11 (11.4)	8 (9.3)	
Urinary tract	13 (7.1)	9 (9.3)	4 (4.6)	
Skin and soft tissue	4 (2.2)	2 (2.1)	2 (2.3)	
Others	35 (19.2)	21 (21.8)	14 (16.2)	
SOFA score at admission (mean, SD)	6.3 (3.2)	5.5 (3.0)	7.1 (3.3)	<0.001*
SOFA score at seven days (mean, SD)	5.8 (3.5)	4.2 (2.7)	6.6 (3.6)	0.003*
GCS at admission (median, IQR)	12 (8-15)	14 (10-15)	11 (7.5-15)	0.019
VIS (median, IQR)	21 (10-50)	10 (10-20)	30 (18-85)	<0.001*
PELOD score (mean, SD)	5.5 (4)	4 (3)	6 (4)	<0.001*
PELOD score categories				0.058
Low <10	158 (86.8)	88 (91.7)	70 (81.4)	
Medium 10-19	23 (12.6)	8 (8.3)	15 (17.4)	
High ≤20	1 (0.5)	0	1 (1.2)	

Table 2 shows the laboratory findings and interventions for patients admitted to the PICU with sepsis. Invasive mechanical ventilation support was required in 48.9% of the whole cohort. Patients in the corticosteroid group required more ventilation support than those in the non-corticosteroid group (72% vs. 28%, respectively), as well as more use of inotropes and vasopressors (76% vs. 34%) and a longer mean prothrombin time (16, SD=7.4 vs. 14, SD=4). The culture was positive in 47.2% of the patients and was caused by a bacterial pathogen in 46.7% of those with positive culture results.

**Table 2:** Descriptive characteristics of laboratory findings and intervention for patients admitted to the PICU with sepsis, stratified by steroid exposure IQR - interquartile range; SD - standard deviation; INR - international normalized ratio; PTT - partial thromboplastin time; PICU - pediatric intensive care unit; PO_2_FIO_2_ - ratio of arterial oxygen partial pressure to fractional inspired oxygen * Significant p-value ** The percentage is taken from those with a positive culture

Variables	All patients (n=182)	Non-corticosteroid group (n=96; 52.7%)	Corticosteroid group (n=86; 47.3%)	p-value
Mechanical ventilation	89 (48.9)	27 (28.1)	62 (72.1)	<0.001*
PO_2_FIO_2_% (mean, SD)	174.6 (104)	190 (108)	156 (96)	0.028*
Admission laboratory results				
Neutrophil count (µL; median, IQR)	6.2 (2.7-12.2)	5.5 (2.9-12.9)	6.5 (2.2-11)	0.667
Hemoglobin level (g/l; mean, SD)	113 (29.2)	116 (28.3)	110 (30)	0.158
Platelet count (platelets per microliter of blood; median, IQR)	242 (137-360)	251 (146-360)	221 (103-367)	0.204
Prothrombin time (seconds; mean, SD)	15 (5.9)	14 (4.0)	16 (7.4)	0.033*
INR (mean, SD)	1.4 (0.99)	1.3 (0.3)	1.65 (1.3)	0.025*
PTT (seconds; median, IQR)	33.4 (29-42)	32.8 (28-41)	33.5 (29.5-45)	0.325
PO_2_FIO_2_% (mean, SD)	174.6 (104)	190 (108)	156 (96)	0.028*
Culture positive				
All patients with positive culture**	86 (47.2)	42 (43.7)	44 (51.1)	0.317
Sputum	33 (18.1)	8 (8.3)	25 (29.1)	<0.001*
Urine	46 (25.2)	22 (22.9)	24 (27.9)	0.439
Blood	63 (34.6)	35 (36.4)	28 (32.5)	0.581
Type of infection				
Bacterial	85 (46.7)	43 (44.7)	42 (48.8)	0.585
Gram-positive	46 (25.2)	20 (20.8)	26 (30,0)	0.145
Gram-negative	39 (21.4)	23 (23.9)	16 (18.6)	0.154
Viral	13 (7.1)	8 (8.3)	5 (5.8)	0.487
Fungal	8 (4.3)	2 (2.0)	6 (7.0)	0.103
Medication received				
Receiving vasopressors and inotropes	99 (54.0)	33 (34.3)	66 (76.7)	<0.001*

Study outcomes

**Figure 1:**
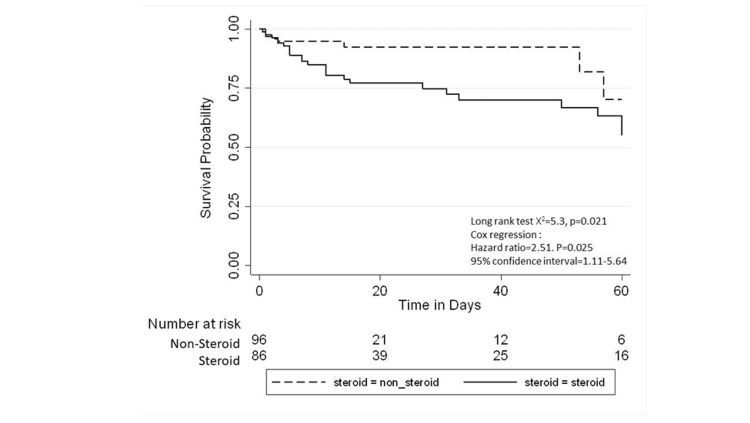
Kaplan-Meier curve of mortality within 60 days of sepsis diagnosis date for patients admitted to the PICU with sepsis, stratified by steroid exposure PICU - pediatric intensive care unit

**Figure 2:**
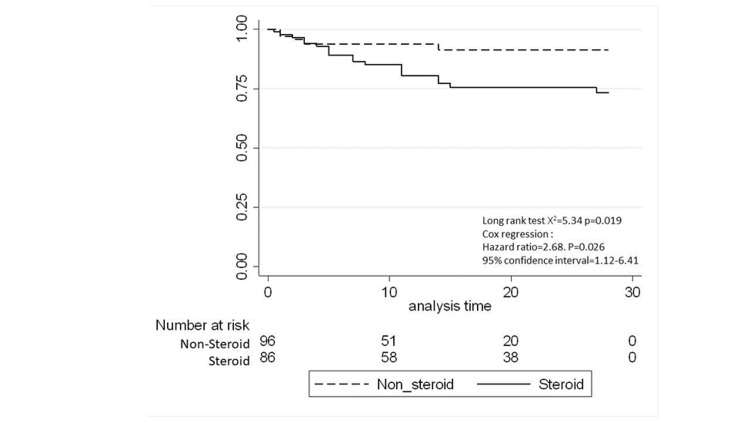
Kaplan-Meier curve of mortality within 28 days of sepsis diagnosis date for patients admitted to the PICU with sepsis, stratified by steroid PICU - pediatric intensive care unit

**Figure 3:**
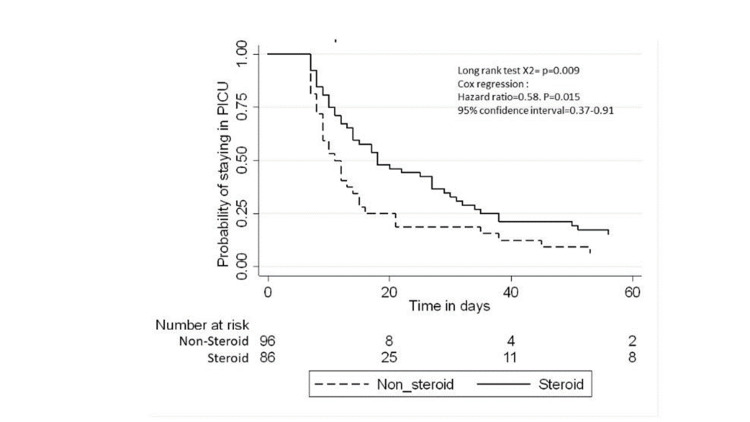
Kaplan-Meier curve of PICU length of stay from sepsis diagnosis date for patients admitted to the PICU with sepsis, stratified by steroid PICU - pediatric intensive care unit

**Figure 4:**
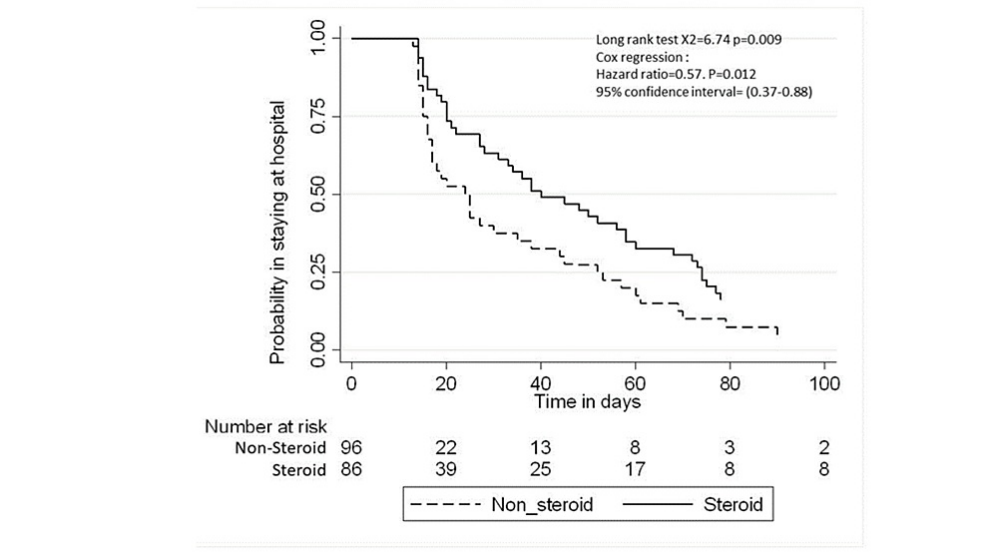
Kaplan-Meier curve of the length of hospital stay from sepsis diagnosis date for patients admitted to the PICU with sepsis, stratified by steroid PICU - pediatric intensive care unit

All-cause mortality was 17.5% among the whole study population and was higher in the corticosteroid group than in the non-corticosteroid group (27% vs. 8%; p=0.001, Table 3). Figure 1 and 2 provides a Kaplan-Meier curve for mortality within 60 days and 28 days of the sepsis diagnosis date for patients admitted to the PICU with sepsis, stratified by steroid exposure. The median PICU stay was six days (IQR: 2-14 days), and the median hospital stay was 12 days (IQR: 7-33 days). The Kaplan-Meier curves for the length of PICU stay and hospital stay are shown in Figures 3 and 4.

**Table 3:** Clinical course and outcomes among patients admitted to the PICU with sepsis IQR - interquartile range; PICU - pediatric intensive care unit

Variables	All patients (n=182)	Non-corticosteroid group (n=96)	Corticosteroid group (n=86)	p-value
All-cause mortality	32 (17.5)	8 (8.3)	24 (27.9)	<0.001*
28-day mortality	26 (14.2)	8 (7.2)	19 (22.1)	<0.004*
Length of stay in PICU in days (median, IQR)	6 (2-14)	4 (2-8)	9 (3-27)	<0.001*
Length of stay in PICU category				< 0001*
≤6 days	98 (53.9)	64 (66.7)	34 (39.5)	
>6 days	84 (46.1)	32 (33.3)	52 (60.5)	
Length of stay in hospital in days (median, IQR)	12 (7-33)	10 (7-18)	16 (8-50)	0.020*
≤12 days	93 (51.1)	56 (58.3)	37 (43.0)	
>12 days	89 (48.9)	40 (41.7)	49 (57.0)	

Overall, the crude mortality rate was higher among patients who received corticosteroids (10.8 cases per 1,000 person-years of follow-up) than in patients in the non-corticosteroid group (5 cases per 1,000 person-years of follow-up; Table 4).

**Table 4:** Risk of mortality and adverse events associated with steroid therapy in patients admitted to the PICU with sepsis HR - hazard ratio; CI - confidence interval; PICU - pediatric intensive care unit; SOFA - Sequential Organ Failure Assessment; PELOD - Pediatric Logistic Organ Dysfunction; VIS - vasoactive-inotropic score ^a^ adjusted for age categories, pre-existing medical conditions, severity score (SOFA, PELOD, VIS), platelet count at admission, and prothrombin time at admission ^b^ adjusted for the use of vasopressors, inotropes, and mechanical ventilation

Outcome	Number of events		Rate of event per 1,000 person-years (95% CI)		Unadjusted HR (95% CI)	p-value	Adjusted HR (95% CI)^a^	p-value	Adjusted HR (95% CI)^b^	p-value
	Non-corticosteroids	Corticosteroids	Non-corticosteroids	Corticosteroids						
All-cause mortality	8	24	5.0 (2.51-0.06)	10.8 (7.30-16.25)	2.50 (1.11-5.64)	0.026	2.61 (0.66-10.28)	0.170	1.44 (0.56-3.68)	0.353
28-day mortality	7	19	5.7 (2.73-12.05)	13.2 (8.47-20.83)	2.62 (1.16-5.89)	0.019*	2.38 (0.43-13.06)	0.318	1.34 (0.50-3.55)	0.551
Length of PICU stay in days										
>6 days	32	52	41.3 (29.23-58.46)	27.8 (21.25-36.60)	0.58 (0.37-0.91)	0.021*	0.35 (0.13-0.98)	0.048	0.62 (0.37-1.02)	0.062
Length of hospital stay in days										
>12 days	40	49	21.5 (15.8-29.3)	14.6 (11.1-19.4)	0.57 (0.37-0.88)	0.012*	0.93 (0.34-2.52)	0.898	0.65 (0.38-1.11)	0.118

The unadjusted Cox regression analysis revealed that the exposed group had an increased risk of mortality compared to the controls, with an HR of 2.50 (95% CI: 1.11-5.64). After adjusting for age categories, pre-existing medical conditions, severity score (SOFA, PELOD, VIS), platelet count, and prothrombin time at admission, no statistical association was found between corticosteroid use and mortality (HR: 2.61; 95% CI: 0.66-10.28). Similarly, after further adjusting for vasopressors, inotropes, and mechanical ventilation, the HR decreased to 1.44 (95% CI: 0.56-3.68). Those who received corticosteroid steroids had 42% less risk of staying in the PICU for more than six days compared to those who did not receive corticosteroids (adjusted HR: 0.35; 95% CI: 0.13-0.98, Table 5).

**Table 5:** Clinical and outcome variables stratified by survival status and within the subset of patients who received steroids GCS - Glasco Coma Scale; SOFA - Sequential Organ Failure Assessment; PELOD - Pediatric Logistic Organ Dysfunction; VIS - vasoactive-inotropic score; PICU - pediatric intensive care unit The percentage in the table is presented as a column percentage. * Significant p-value ** The percentage was calculated from those who had pre-existing conditions at admission

Variables	All patients			Patients who received steroids		
	Survived (n=150, 82.4%)	Non-survived (n=32, 17.5%)	p-value	Survived (n=62, 72%)	Non-survived (n=24, 28%)	p-value
Pre-existing medical condition at admission			0.840			0.352
No	35 (23.3)	8 (25.0)		22 (35.4)	6 (25.0)	
Yes	115 (76.7)	24 (75.0)		40 (64.5)	18 (75.0)	
Comorbidity system among pre-existing medical condition at admission**						
Neurology disease	41 (27.3)	7 (21.8)	0.525	13 (20.9)	5 (20.8)	0.620
Genetic/metabolic disease	30 (20)	9 (28.1)	0.309	9 (14.5)	6 (25.0)	0.250
Cardiovascular disease	3 (2.0)	2 (6.2)	0.212	1 (1.6)	1 (4.1)	0.483
Pulmonary disease	10 (6.6)	4 (12.5)	0.261	8 (12.9)	4 (16.6)	0.443
Malignancy	9 (6.0)	1 (3.1)	0.448	1 (1.6)	1 (4.1)	0.483
Genitourinary	8 (5.3)	-	0.206	2 (3.2)	-	0.517
Gastrointestinal	9 (6.0)	1 (3.1)	0.517	3 (4.8)	1 (4.1)	0.894
Other	5 (3.3)	-	0.273	3 (4.8)	-	0.370
Culture positive						
Sputum	27 (18.0)	6 (18.7)	0.920	19 (30.6)	6 (25.0)	0.605
SOFA at admission (mean, SD)	5.8 (2.9)	8.8 (3.7)	<0.001*	6.5 (3.1)	8.78 (3.4)	0.005*
SOFA at seven days (mean, SD)	4.96 (3.1)	9.4 (3.2)	<0.001*	5.54 (3.1)	9.9 (3.0)	<0.001*
GCS at admission (median, IQR)	8 (5-15)	13 (9-15)	0.017	8 (3-15)	8 (3-14)	0.260
VIS (median, IQR)	20 (10-42)	35 (18-175)	0.001*	25 (10-50)	85 (25-200)	0.003*
PELOD score (mean, SD)	4.9 (3.6)	8 (5.2)	0.001*	5.9 (3.6)	8.3 (5.5)	0.021*
Length of PICU stay (median, IQR)	6 (2–13)	6.5 (3-17.5)	0.385	9.5 (3-27)	6.5 (5-24)	0.992

## Discussion

The latest guidelines recommend the use of corticosteroids for patients with fluid-resistant and catecholamine-resistant septic shock [10], but the effect of corticosteroid administration on mortality and length of stay remains unclear, so a number of studies have investigated the risk of mortality among pediatric patients admitted to the ICU with a diagnosis of sepsis and treated with corticosteroids.

The present study found no statistically significant difference between those who received and those who did not receive corticosteroids in terms of mortality and length of stay (p=0.353 and p=0.492, respectively), which is consistent with several studies [6-11]. A study with over 400 patients found no significant difference in survival rate [6], and another large study, which gathered data from 18 pediatric centers in multiple countries, found that the administration of corticosteroids was not associated with better outcomes [11]. By contrast, a study of over 6,000 patients found that the risk of mortality was significantly increased by corticosteroids, but the researchers were unable to determine illness severity scores [8]. Our study determined multiple illness-severity scores (e.g., SOFA and PELOED) in the PICU on the first and seventh days after admission.

Regarding illness severity, the mean SOFA score of those who received corticosteroids in our study is 7.1, while it is 5.5 for those who did not, and the difference is statistically significant (p<0.001). By contrast, a retrospective study of 477 children with severe sepsis concluded that patients who received corticosteroids exhibited similar illness severity in comparison to those who did not. That study also reported that no significant improvement in outcome was found in patients who received corticosteroids [11]. Furthermore, the mean PELOD score in our study is six in the corticosteroid group, which is exactly the value found in a study by Menon et al. [6].

A survey of pediatric intensivists in Canada regarding the use of corticosteroids in critically ill septic patients revealed that 51% of them would empirically administer corticosteroids upon confirmed septic shock diagnosis [12]. Our study found that 47% of the patients (n=86) received corticosteroids.

The patients in our study who received corticosteroids required vasopressors more than those who did not (74.4%), and the difference is statistically significant (p<0.001), a finding similar to that of Atkinson et al., whose study reports a greater requirement of vasopressor agents during the first seven days in the corticosteroid group, with a significant p-value [13]. Those authors found no difference in terms of the type of infection, which is also consistent with our findings.

Regarding mechanical ventilation, 72% of those who received corticosteroids required mechanical ventilation in our study, with a significant p-value, while only 28.1% were ventilated in the control group. A randomized control trial (RCT) in seven tertiary ICUs in Canada found that 61.5% of patients required mechanical ventilation, but no statistical difference was found between the two groups [6].

In terms of length of stay, we measured both the total length of stay and stay in the PICU. We found that the corticosteroid group stayed in the PICU for a median of nine days, which is slightly higher than the 8.4 days reported in another study [6]. In our study, the median total length of hospital stay is 16 days for those who received corticosteroids, while in the other group, the median is 10 days. However, those who received corticosteroids had 42% less risk of staying in the PICU for over six days than those who did not receive steroids (HR: 0.35; 95% CI: 0.13-0.98); the p-value was statistically significant.

Limitations

The exact timing of the administration of medications was not analyzed, which may be important, and some recent studies have stratified patients based on the time of administration, either early (within nine hours of starting vasopressors) or late [14,15]. Thus, our data may be affected due to time variability. As this study was conducted in a single center, a larger sample size could not be obtained. Finally, further RCTs with larger sample sizes are needed to complement our findings.

## Conclusions

After adjusting for baseline characteristics, severity scores, and medical intervention, no association was found between receiving corticosteroids and mortality (p=0.492), but patients who received corticosteroids had less risk of a prolonged stay in the PICU than those who did not (HR: 0.35; 95% CI: 0.13-0.98).
